# Self‐Assembly of Off‐Target Peptide Sequences: Implications for the Design of Soft Materials

**DOI:** 10.1002/smll.202507714

**Published:** 2025-09-02

**Authors:** Yanyao Wang, Ravi R. Sonani, Libby Marshall, Simona Bianco, Karen Marshall, Alice Pincham, Honghui Yang, Louise C. Serpell, Annela M. Seddon, Edward H. Egelman, Andrew R. Thomson, Dave J. Adams

**Affiliations:** ^1^ Department of Applied Chemistry Xi'an Jiaotong University Xi'an 710049 P. R. China; ^2^ School of Chemistry University of Glasgow Glasgow G12 8QQ UK; ^3^ Department of Biochemistry and Molecular Genetics University of Virginia Charlottesville VA 22903 USA; ^4^ Sussex Neuroscience School of Life Sciences University of Sussex Brighton BN1 9QG UK; ^5^ School of Physics HH Wills Physics Laboratory University of Bristol Bristol BS8 1TL UK

**Keywords:** cryoEM, design, peptide, SAXS, self‐assembly

## Abstract

The self‐assembly of short peptides into defined nanostructures is one method for preparing soft materials and gels. Indeed, many useful materials can be prepared by the self‐assembly of oligopeptides. The design rules around such peptides are relatively established, and they assume well‐defined and pure materials. In many cases, however, the purity of the peptide is less than 95%, and the ability of likely impurities to self‐assemble is an open question. Here, the self‐assembly of the gel‐forming octapeptide FEFEFKFK and two analogues, EFEFKFK and FEFEfKFK, is discussed to examine the effect of an amino acid deletion and of epimerization at one position. Both the truncated peptide and epimerized peptide can still form gels. Mixing these peptides with the parent FEFEFKFK leads to the formation of new, but different, self‐assembled structures. This has direct implications for the understanding of the necessary design rules for self‐assembly regarding the influence of potential impurity in these systems, as well as demonstrating that much remains to be learned about sequence to structure relationships in self‐assembling peptide systems.

## Graphical abstract

For an ionic complementary peptide, both a truncated peptide and an epimerized peptide can still form gels. Mixing these peptides with the parent FEFEFKFK leads to the formation of new, but different, self‐assembled structures.

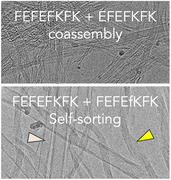

## 1 Introduction

The self‐assembly of short peptides can be used to form useful materials, including gels for cell culture and tissue engineering, optoelectronics, drug delivery, pressure‐sensitive materials, and sensors.^[^ ^1^, ^2^, ^3^ ^]^ A number of these materials are now commercialized.^[^ ^4^, ^5^ ^]^ A well‐known strategy for forming peptide‐based materials involves the double layers of β‐strands, in which repeats of amino acids with a high β‐strand forming potential are used, usually by alternating polar and hydrophobic residues in the sequence.^[^ ^6^, ^7^ ^]^ Arguably starting the field of peptide self‐assembly to make a priori defined materials, Zhang et al. showed in the 1990s that such amphipathic peptides assemble into structures with hydrophobic and hydrophilic faces on opposite sides;^[^ ^8^ ^]^ the hydrophobic faces collapse to form tapes leading to cross β‐sheet formation. Examples of such peptides include (using the one letter amino acid codes) FEFEFKFK,^[^ ^9^, ^10^, ^11^, ^12^, ^13^ ^]^ FEFKFEFK,^[^ ^14^ ^]^ KFFEEAAAKKFFEE^[^ ^15^ ^]^ and (RA)4(DA)4. Hydrophobic interactions between nonpolar residues, as well as salt bridges between complementary charged amino acids, for example between E and K residues, direct peptide‐peptide interactions to give specific, programmed structures. There is a surprising lack of new designs in this area, and as new techniques become available, it has been shown that some of the assumptions of packing that these rules are based on are not necessarily correct.^[^ ^16^ ^]^ Almost all examples of such self‐assembling short peptides are close analogues of peptides reported in the late 1990s.^[^ ^17^ ^]^ Peptides as short as 4 residues^[^ ^18^ ^]^ and as long as 16 residues^[^ ^19^ ^]^ have been shown to form similar fibrillar structures. The pattern of charge in ionic complementary structures has been investigated.^[^ ^20^, ^21^, ^22^ ^]^ Algorithms used to predict and understand the structure of amyloid have been used to gain insight here.^[^ ^23^ ^]^ Block heterochiral analogues have been examined, for example, with 4 L‐amino acids, followed by 4 D‐amino acids; here, helical tapes are formed as opposed to twisted tapes.^[^ ^24^ ^]^ Despite numerous empirical studies, understanding the relationship between peptide sequence and material morphology is still a largely unmet challenge. Atomistic structural information is difficult to obtain experimentally, and modelling methods such as molecular dynamics simulations are not generally suitable for identifying minimum energy structures.^[^ ^25^, ^26^ ^]^ The application of cryogenic‐electron microscopy (Cryo‐EM) to peptide assemblies has changed this landscape, and near‐atomic resolution of such assemblies is now becoming nearly routine.^[^ ^14^, ^27^, ^28^ ^]^

These systems are generally studied using synthetic peptides. Though high synthetic purities are accessible, these materials inevitably contain some peptide impurities related to the target sequence. An interesting question, therefore, is how robust these designs are to the presence of similar, but off‐target peptide sequences. Impurities are generally assumed not to contribute to self‐assembly. Surprisingly, especially considering that epimerization is a likely side reaction in peptide synthesis, there is little data on examples where a single amino acid is of opposite chirality to the remainder. Impurities may have a significant effect on the self‐assembly process as they could co‐assemble with the peptide, affecting the molecular packing, for example, but there are to our knowledge no specific studies examining this.

**Figure 1:**
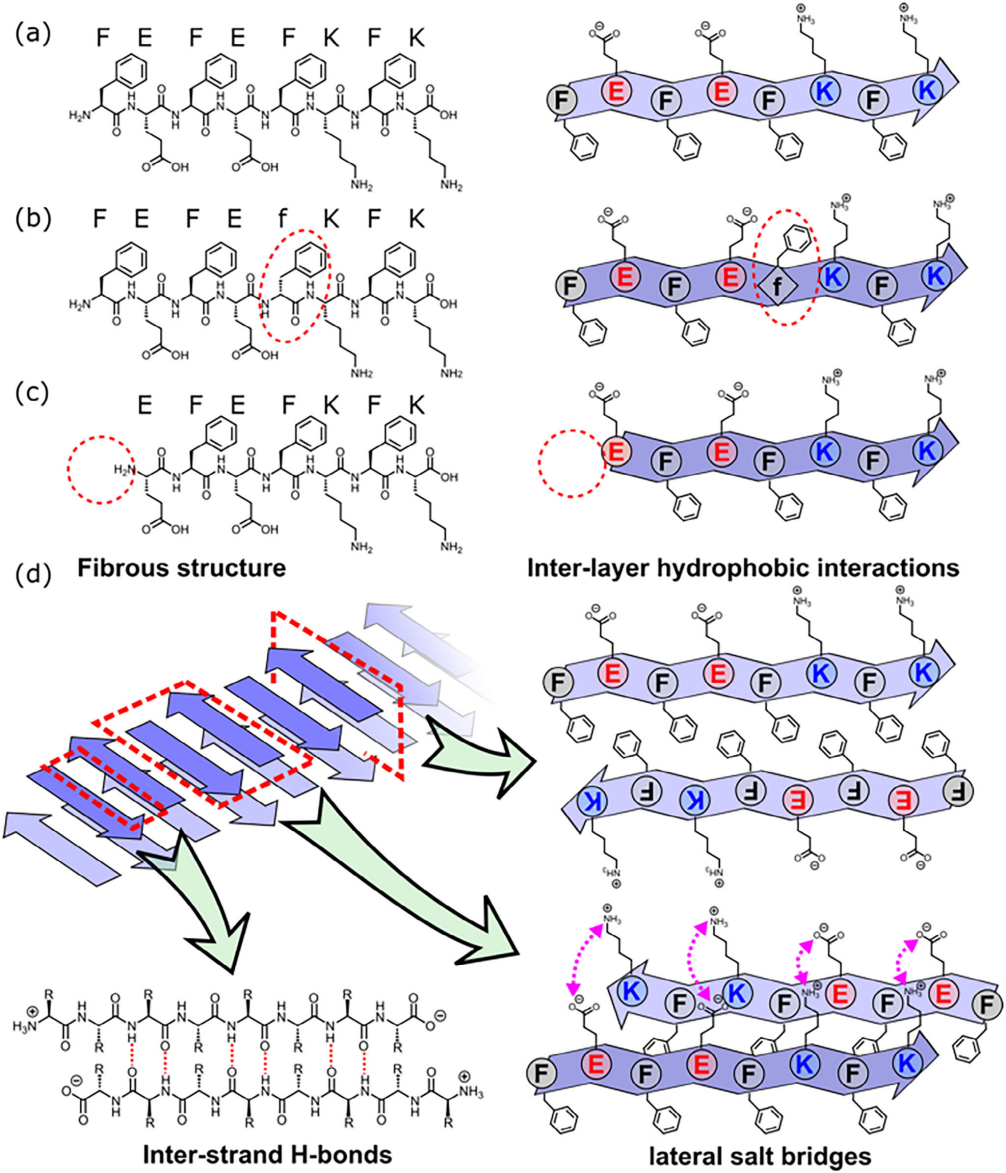
Chemical structures and cartoon representations of a) FEFEFKFK, b) FEFEfKFK, and c) EFEfKFK. d) Cartoon of the self‐assembly of FEFEFKFK leading to fiber formation and gelation based on the literature assignment of antiparallel β‐sheets.

Here, we focus on FEFEFKFK (**Figure** **1**a), and evaluate the self‐assembly capacity of peptides representing, separately, a sequence truncation and point epimerization. As mentioned above, this peptide is an example of an ionic complementary peptide with alternating charged (hydrophobic and hydrophilic) amino acid residues and self‐assembles into fibers that entangle to form a gel. Fiber formation is thought to occur due to the formation of anti‐parallel β‐sheets (Figure 1d), leading to a hydrophobic face where all F residues are on one side.^[^ ^10^, ^11^, ^12^ ^]^ Hydrophobic collapse leads to fiber formation, combined with interactions between amino acids with different charges. From this perspective, it might be expected that deletion of one of the amino acids or epimerization at one position would lead to significantly perturbed packing and hence have a (possibly adverse) effect on the self‐assembly. However, here, we demonstrate that a sequence with a deletion, EFEFKFK (Figure 1c), can form gels despite the reduction in *π*‐interactions possible.^[^ ^15^, ^29^ ^]^ Similarly, the epimerization of a single residue (FEFEfKFK, Figure 1b) does not prevent self‐assembly into fibrillar structures and gels. Mixing of the peptides leads to interesting new structures. This has direct implications for our understanding of the impact of peptide purity issues in these systems, as well as opening interesting new design possibilities.

## 2 Results and Discussion

FEFEFKFK was prepared with a purity of ≈92%; typical for this peptide^[^ ^12^ ^]^ (Figure S1 and Table S1, Supporting Information). LC‐MS analysis of this peptide indicates the presence of small amounts of single and double amino acid deletions, corresponding to loss of F, E, and K, with E and K deletions being of very similar mass. No higher mass impurities were observed, as could arise from residual protecting groups or extra amino acids inserted in the sequence. We therefore focused on sequences with deletions and where amino acids have epimerized during the synthesis. Hence, we examined two analogues: EFEFKFK (Figure 1c), where the peptide has been truncated by one amino acid, and FEFEfKFK, where one phenylalanine is epimerized to the D‐enantiomer (Figure 1b). We chose this epimerization position on the basis that it is the central hydrophobic residue, likely to have the greatest impact on the packing within the core of the fiber. In both cases, we expected that differences in the packing of the peptides would affect the stability of protofilaments and, therefore, how such protofilaments would associate into larger structures and ultimately gels. EFEFKFK and FEFEfKFK were prepared with purities of ≈93% and 97% respectively (Table S1, Supporting Information).

We investigated the self‐assembly and gelation of all three of these single components systems. In addition, from the perspective of either the truncated peptide or epimerized peptide representing typical impurities, we investigated binary mixtures of the peptides. In all cases, we used the established heat‐cool method of self‐assembly at pH 7.4. Typically, in binary systems, two main possibilities exist.^[^ ^30^, ^31^, ^32^ ^]^ Co‐assembled structures could be formed, whereby each self‐assembled structure contains both components present. Alternatively, self‐sorted systems could form, whereby any individual self‐assembled structure would contain only one of the components.

**Figure 2:**
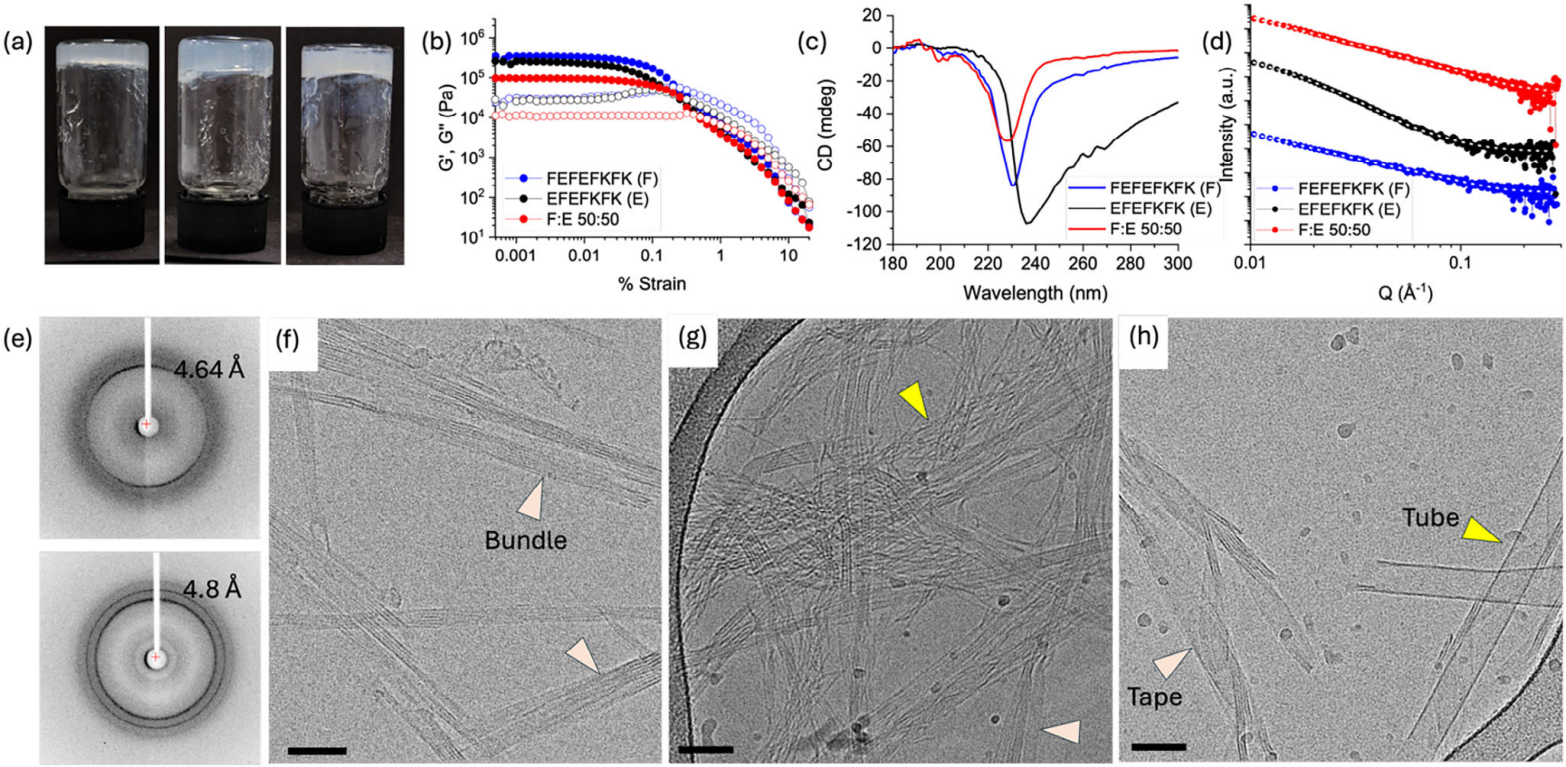
a) Photograph of gels formed from (left) EFEFKFK, (middle) a 50:50 mixture of FEFEFKFK and EFEFKFK, and (right) FEFEFKFK; b) Strain sweep for gels formed from (black) EFEFKFK, (red) a 50:50 mixture of FEFEFKFK and EFEFKFK, and (blue) FEFEFKFK. G′ is shown as full circles and G″ as open circles. Rheological data presented were obtained from single sample measurements. c) Overlay of CD data for (black) EFEFKFK, (red) a 50:50 mixture of FEFEFKFK and EFEFKFK and (blue) FEFEFKFK; d) SAXS data (circles) and fit (white line) for gels formed from (black) EFEFKFK, (red) a 50:50 mixture of FEFEFKFK and EFEFKFK and (blue) FEFEFKFK (full details of the fits can be found in Figure S6,S7 and S9, Supporting Information). All measurements were corrected for transmission and absolute intensity and had the solvent background and empty capillary scattering subtracted before processing. The data were reduced using SAXSGUI. Error bars are generated during data correction and processing. SAXS data are stacked for ease of comparison, and each dataset was offset using a multiplier. Circular dichroism spectra were recorded in quadruplicate and averaged. These were recorded as a single measurement on the same sample. The data represent the averaged spectra. e) fXRD shows (top) a ring with a spacing of 0.46 nm for EFEFKFK and (bottom) rings with spacings at 0.48 nm for a 50:50 mixture of FEFEFKFK and EFEFKFK; f) Cryo‐EM micrograph of a EFEFKFK gel. Bundles of fibers are indicated by arrowheads. g) Cryo‐EM micrograph of a 50:50 mixture of FEFEFKFK and EFEFKFK showing laterally associated fibers in two different manners, indicated by different color arrowheads; h) Cryo‐EM micrograph of a FEFEFKFK gel. Tapes and tubes are indicated by arrowheads. For all cryo‐EM micrographs, scale bars = 50 nm. In all cases, the gels were formed at 20 mg mL^−1^.

For EFEFKFK alone, gels are formed above a concentration of 12 mg mL^−1^. Gels formed at a concentration of 20 mg mL^−1^ (**Figure** **2**a) have very similar rheological properties to the parent peptide with a storage modulus (G′) of 2.8 × 10^5^ Pa and a loss modulus (G″) of 2.8 × 10^4^ Pa (Figure 2b; Figure S2, Supporting Information; at this concentration, FEFEFKFK gels exhibited a G′ of 3.5 × 10^5^ Pa and a G″ of 2.6 × 10^4^ Pa). The gels are frequency independent (Figure S2, Supporting Information) and break at low strain, typical for such peptide gels. Previous work with FEFEFKFK has suggested an antiparallel β‐sheet structure.^[^ ^12^, ^19^ ^]^ CD shows a minimum at 235 nm, similar to that of FEFEFKFK at 231 nm, but with a significantly broader shoulder than the parent peptide (Figure 2c). Fiber X‐ray diffraction (fXRD) of an air‐dried stalk composed of a bundle of fibrils shows diffraction at 4.64 Å, characteristic of hydrogen bonding distance between β‐strands arranged in a cross‐β structure (Figure S3, Supporting Information). fXRD of a dried stalk of EFEFKFK shows similar diffraction at 4.64 Å (Figure 2e). IR shows the presence of peaks at 1614, 1618, and 1692 cm^−1^, correlating with β‐sheet formation (Figure S4, Supporting Information). Truncation does not therefore, seem to fundamentally change the molecular packing in the self‐assembled structures formed.

To determine whether the absolute self‐assembled structures are different, we used TEM and SAXS. TEM shows the presence of bundles of fibers (Figure S11b, Supporting Information). These can be resolved well using cryo‐EM (Figure 2f). The SAXS data for the gel phase are best fit to a flexible elliptical cylinder model combined with a power law, with a radius of 5.7 nm and an axis ratio of 2.5 (Figure 2d; Figure S7, Supporting Information). On diluting the sample, cryo‐EM (Figure S12, Supporting Information) shows the presence of filaments, suggesting that these are primary structures that are being detected by SAXS. In comparison, cryo‐EM analysis for FEFEFKFK shows the presence of tapes and tubes (Figure 2h). The SAXS data from the gel of FEFEFKFK are best fit to a cylinder model combined with a power law (Figure 2d; Figure S6, Supporting Information), giving a radius of 2 nm (cryo‐EM of a diluted sample shows objects with such a diameter, Figure S12, Supporting Information). Hence, the truncated peptide forms filaments of similar dimensions to that formed by FEFEFKFK, but with a different cross‐sectional shape that further assembles into different aggregates.

For mixtures of FEFEFKFK and EFEFKFK, at a ratio of 50:50, the rheological values of the gel show a decrease in stiffness compared to the gel formed from EFEFKFK alone, with similar rheological behavior to FEFEFKFK alone (Figure 2b). fXRD of a dried stalk again shows diffraction at 4.8 Å, indicative of cross β‐structure formation (Figure 2e). The CD spectrum is similar in shape to FEFEFKFK, but blue shifted (Figure 2c). The SAXS data are similar to that of FEFEFKFK alone and best fit to a cylinder model combined with a power law with a radius of 2.1 nm (Figure 2d; Figure S9, Supporting Information). TEM shows the presence of aggregated fibers (Figure S11d, Supporting Information). Cryo‐EM of the mixture shows the presence of relatively ordered laterally associated fibers (Figure 2g). From all these data, we suggest that the two peptides are co‐assembling, leading to structures being formed that are packed in a similar manner to the parent peptide. Interestingly, gels are formed across a wide composition range for mixtures of FEFEFKFK and EFEFKFK; gels are formed at most ratios examined, but not at ratios of 80:20, 70:30 and 60:40. Indeed, stiff gels are formed even at a ratio of 90:10, which shows that impurity levels at this degree do not lead to the system being unable to form gels (Figure S13, Supporting Information). Here, we use a total peptide concentration of 20 mg/mL, so the ratios at which no gels are formed are at a concentration of FEFEFKFK above the minimum gelation concentration; this implies that the EFEFKFK is perturbing the self‐assembly by co‐assembly. The CD spectra show a gradual shift in the peak minimum depending on the composition (Figure S13, Supporting Information). This leads to an important point; were such truncated peptides present in the system due to issues during synthesis, for example, it is not clear that this would be obvious from much of the data, even up to very high concentrations of EFEFKFK. Indeed, a small concentration might not be detected, but could still affect the macroscopic properties and, for example, lead to reproducibility issues.

**Figure 3:**
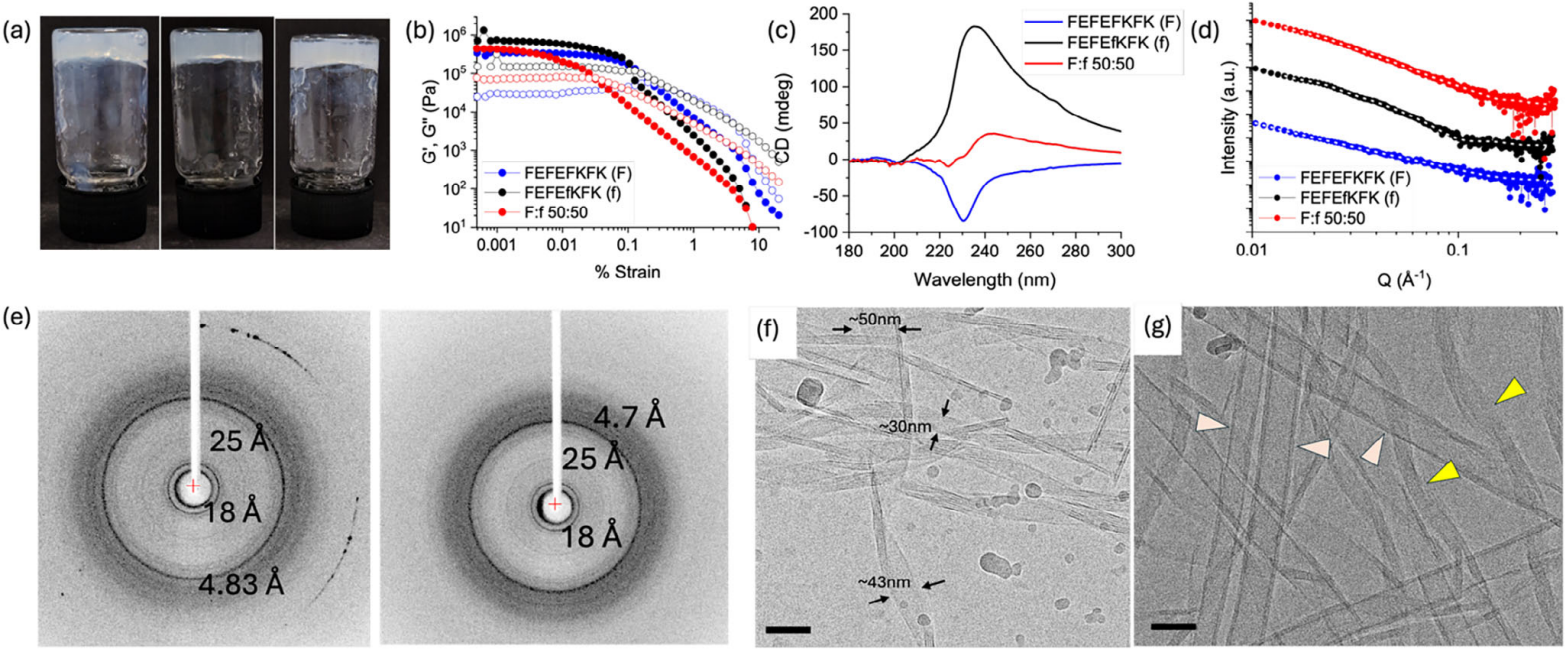
a) Photograph of gels formed from (left) FEFEfKFK, (middle) a 50:50 mixture of FEFEFKFK and FEFEfKFK, and (right) FEFEFKFK; b) Strain sweep for gels formed from (black) FEFEfKFK, (red) a 50:50 mixture of FEFEFKFK and FEFEfKFK, and (blue) FEFEFKFK. G′ is shown as full circles and G″ as open circles. Rheological data presented were obtained from single sample measurements. c) Overlay of CD data for (black) FEFEfKFK, (red) a 50:50 mixture of FEFEFKFK and FEFEfKFK, and (blue) FEFEFKFK; Circular dichroism spectra were recorded in quadruplicate and averaged. These were recorded as a single measurement on the same sample. The data represent the averaged spectra. d) SAXS data (circles) and fit (white line) for gels formed from (black) FEFEfKFK, (red) a 50:50 mixture of FEFEFKFK and FEFEfKFK, and (blue) FEFEFKFK (full details of the fits can be found in Figures S6, S8, and S10, Supporting Information). All measurements were corrected for transmission and absolute intensity and had the solvent background and empty capillary scattering subtracted before processing. The data were reduced using SAXSGUI. Error bars are generated during data correction and processing. SAXS data are stacked for ease of comparison, and each dataset was offset using a multiplier.; e) fXRD shows (left) ring with a spacing of 0.48, 1.8, and 2.5 nm for FEFEfKFK and (right) rings with spacings at 0.48, 1.8, and 2.5 nm for a 50:50 mixture of FEFEFKFK and FEFEfKFK; f) Cryo‐EM micrograph of a FEFEfKFK gel showing different size tapes; g) Cryo‐EM micrograph of a 50:50 mixture of FEFEFKFK and FEFEfKFK showing tapes (yellow arrowheads) and tubes (orange arrowheads). For all cryo‐EM micrographs, scale bars = 50 nm. In all cases, the gels were formed at 20 mg mL^−1^.

Moving to FEFEfKFK, it might be expected that the epimerized peptide would be unable to form well‐defined nanostructures depending on whether the _D_‐phenylalanine packs such that the bilayer is disrupted or not (Figure 1d). Perhaps surprisingly, therefore, this peptide also forms gels above a concentration of 8 mg mL^−1^ at pH 7.4. At a concentration of 20 mg mL^−1^ (**Figure** **3**a), the gels are stiffer than those formed by the parent peptide, with a G′ of 7.4 × 10^5^ Pa and a G″ of 1.6 × 10^5^ Pa (Figure 3b). The gels are again frequency independent (Figure S2, Supporting Information). fXRD of a dried stalk again shows diffraction at 4.83 Å, indicative of cross β‐structure formation (Figure 3e). Interestingly, the addition of the equatorials at 25 and 18 Å that are measurable here suggests that there is a regular ordering in the packing. Despite only one amino acid being changed to the _D_‐isomer, CD shows an inversion of signal compared to the parent peptide, with a positive peak at 227 nm (Figure 3c). This implies that the structures formed have an opposite twist to that of the parent peptide. IR spectroscopy shows a peak at 1617 and a peak at 1680 cm^−1^ (Figure S4, Supporting Information). The SAXS data for the gel phase are best fit to a hollow cylinder model combined with a power law with a radius of 12.7 nm and a thickness of 5.8 nm (Figure 3d; Figure S8, Supporting Information). TEM shows the presence of tubes, which can be seen to be formed by wrapping up of tapes (Figure S11c, Supporting Information). These tapes can be imaged by cryo‐EM with polydisperse thicknesses. The lateral association of fibers to form these tapes can be seen in the cryo‐EM images (Figure 3f). Overall, self‐assembly into defined nanostructures is still possible despite the epimerized amino acid; however, a very different morphology is adopted compared to FEFEFKFK (Figure 2h).

A gel is formed from a 50:50 mixture of FEFEFKFK with FEFEfKFK (Figure 3a), with a G′ of 4.5 × 10^5^ Pa and a G″ of 7.2 × 10^4^ Pa (Figure 3b). The CD spectrum of the mixture is a composite of that of pure FEFEFKFK and pure FEFEfKFK, implying that self‐sorting is occurring (Figure 3c). This is also suggested by the fXRD where the equatorials at 25 and 18 Å present in FEFEfKFK alone are also detected here, in addition to the expected diffraction at 4.7 Å present in both pure peptides (Figure 3e). TEM shows the presence of tubes and helical tapes (Figure S11e, Supporting Information). These are also imaged by cryo‐EM, which shows tapes and tubes having multi‐layer walls (Figure 3g). The SAXS data are best fit to a model combining a hollow cylinder, a cylinder, and a power law, i.e., a combination of the models required for the individual components (Figure 3d; Figure S10, Supporting Information). As such, it appears that the mixture of FEFEFKFK and FEFEfKFK undergoes self‐sorting.

Gels are formed at all compositions of FEFEFFK and FEFEfKFK examined. The values of G′ and G″ vary across the composition, with gels at a ratio of 90:10 having a lower G′ than the rest, and the 30:70 ratio having the highest G′. As for the EFEFKFK system described above, stiff gels can be formed even in the presence of a significant amount of the FEFEfKFK as compared to the parent peptide (Figure S13, Supporting Information). Self‐sorting is prevalent across the whole composition range as shown by the CD spectra being a linear combination of the spectra for each peptide at the concentration used (Figure S13, Supporting Information).

## 3 Conclusion

We have therefore shown that for ionic complementary peptides based around the sequence FEFEFKFK, a sequence with a deletion, EFEFKFK can form gels despite the reduction in *π*‐interactions possible. Similarly, the epimerization of the single residue, FEFEfKFK, does not prevent self‐assembly into fibrillar structures and gels. Mixing of the peptides leads to interesting new structures, in one case, co‐assembly occurs, and in the other self‐sorting occurs.

There are several interesting implications of these data. First, gels can be formed by co‐assembled or self‐sorted mixtures with rheological values that are not significantly different from gels formed from the pure components. This implies that, at least from the perspective of gel formation, peptide systems can be tolerant of significant levels of impurities. Our results show that off‐target peptides can be competent to self‐assemble with comparable efficiency to the intended sequence. We therefore caution the community to consider the nature of the impurities present in peptide materials as well as the absolute percentage purity. Our data illustrate that anomalous results could plausibly arise from self‐assembly of off‐target sequences present as an impurity in a given sample. Indeed, there are several examples in the literature of purity levels of ≈90% being able to form gels, which agrees with our observations here.

## Conflict of Interest

The authors declare no conflict of interest.

## Supporting information



Supporting Information

## Data Availability

The data that support the findings of this study are available from the corresponding author upon reasonable request.
