# Supplementary material for: Self‐Assembly of Off‐Target Peptide Sequences: Implications for the Design of Soft Materials
Source: Small. 2025 Sep 2;21(42):e07714. doi: 10.1002/smll.202507714 (PMC12548000; doi:10.1002/smll.202507714)
Supplement: Supplementary file 1 — Supporting Information [file SMLL-21-e07714-s001.docx]

Self-Assembly of Off-Target Peptide Sequences: Implications for the Design of Soft Materials

Yanyao Wang,^1,2^ Ravi R. Sonani,^3^ Libby Marshall,^2^ Simona Bianco,^2^ Karen Marshall,^4^ Alice Pincham,^5^ Honghui Yang,^1^ Louise C. Serpell,^4^ Annela M. Seddon,^5^ Edward H. Egelman,^3^ Andrew R. Thomson^2,^* and Dave J. Adams^2,^*

1. Department of Applied Chemistry, School of Chemistry, Xi’an Jiaotong University, Xi’an 710049, People’s Republic of China

2. School of Chemistry, University of Glasgow, Glasgow, G12 8QQ, U.K.

3. Department of Biochemistry and Molecular Genetics, University of Virginia, Charlottesville, VA 22903, U.S.A.

4. Sussex Neuroscience, School of Life Sciences, University of Sussex, Falmer, BN1 9QG, U.K.

5. School of Physics, HH Wills Physics Laboratory, University of Bristol, Bristol BS8 1TL, U.K.

**Supporting Information**

**Experimental methods**

**Peptide synthesis.** All chemicals were purchased from Sigma-Aldrich or Fluorochem Ltd and used as received. Deionized water was employed in all experiments. All the peptides were synthesized by standard solid-phase peptide synthesis (SPPS) methods on a 0.5 mmol scale using a CEM Liberty Blue peptide synthesis instrument. DIC/Oxyma Pure activation chemistry was used. Cleavage from the resin and deprotection of the side groups was carried out using a mix of 95% TFA, 2.5% water and 2.5% TIPS (10 ml total volume) that was added to the resin and it was mixed gently for 2 h. The TFA solution was reduced to 2 mL under a flow of nitrogen. Crude peptides were precipitated with diethyl ether (45 mL) at 0 °C. The solid was recovered by centrifugation and redissolved in 1:1 acetonitrile:water before freeze-drying to yield crude peptides as white solids. HPLC and mass spectrometry were used to confirm the peptide structure and estimate purity.

**Gel preparation.** To prepare gel samples, single peptides or a mixture of peptides were weighed in a 10.5 mL glass vial, followed by addition of deionized water. The mixture then stirred by hand and stood in an ultrasonic bath (Grant XUBA3, 200 watt) at room temperature for 10 mins to obtain a fully dissolved solution. The pH of the initial sample solution was measured to be in the range of 2~3 depending on the peptide concentration due to the presence of residual of TFA. The pH of the solution was adjusted to 7.4 ± 0.2 using 5 M NaOH. To homogenize the solution after addition of base and to minimize the presence of bubbles, the solution was stirred by hand and then heated to 85ºC for 3 hours. Then, the solutions were left to stand quiescently on the bench for at least 16 hours before measurements.

**Analytical HPLC for designed sequences.** Analytical HPLC was performed on SHIMADZU CBM-20A series HPLC systems using a Phenomenex Gemini 5 μm particle size, 110 Å pore size, C18 column of dimensions 150 × 2 mm. Chromatograms were monitored at 214 and 280 nm. Gradients were 10 to 60% acetonitrile in water (each containing 0.1% TFA) for 20 min.

**LC Mass spectrometry.** Mass spectra were collected on an Agilent 1260 Infinity II mass spectrometer operating in positive-ion reflector mode. Phenomenex Gemini 5 μm C18 110 Å, LC Column 150 x 2 mm was employed with a gradient of 10-60% acetonitrile in water for 20 mins. Masses quoted are for the monoisotopic mass as the singly protonated species. Masses were measured to 0.1% accuracy.

**pH measurement.** FC200 pH probe with a 6 mm × 10 mm conical tip from Hanna instruments was used throughout the experiment. The pH meter with an accuracy of ± 0.1 was calibrated by pH 4.01, 7.01 and 10.01 buffer solutions before measurement. The probe was rinsed with deionized water between measurements.

**Small angle X-Ray scattering.** SAXS data were collected on a Ganesha 300XL instrument (Xenocs). SAXS data were collected at room temperature over a Q range of 0.007 – 0.25 Å^-1^ for an exposure time of 3600 seconds. All measurements were corrected for transmission and absolute intensity and had the solvent background and empty capillary scattering subtracted before processing. Data were reduced using SAXSGUI, and model fits were performed using SASView 5.0.4.^1^ Error bars are generated during data correction and processing.

SAXS data presented in Figures 2 and 3 were stacked for ease of comparison and each dataset was offset using a multiplier.

Gel samples were transferred by spatula from vials to quartz capillaries with a 1.5 mm internal diameter. Due to the viscosity of the gels, the capillaries containing the gels were centrifuged in 2000 rad/s for 1 min. The capillaries were then sealed with glue prior to vacuum for 1 hour (Araldite Rapid 15ml x 2 Tubes Epoxy, glue was dried overnight and exposed to vacuum for 1 hour before measurement).

**Circular Dichroism.** Circular dichroism (CD) was measured using a Chirascan VX CD spectrometer (Applied Photophysics Limited, U.K.). The gel samples were transferred from glass vials to a quartz cell with a 0.01 mm path length. The parameters were set as following: 25℃, scanning mode, continuous; scanning speed, 120 nm/min and bandwidth, 1 nm, spectra ranging from 180 to 300 nm or 180 nm to 500 nm at a 2 min interval. All CD data were collected as ellipticity and recorded in millidegree (mdeg). Absorbance and HV spectra were recorded concomitantly with CD spectra (shown in Figure S4 below). All spectra were recorded in quadruplicate and averaged. These were recorded as a single measurement on the same sample.

**Rheology.** All rheological measurements were carried out using an Anton Paar Physica MCR 301 rheometer at 25 °C. Strain and frequency sweeps were performed in triplicate, using flat and parallel geometry (PP12.5) with a gap height of 15~16 mm. The sample vials were put in a custom-made holder that was fixed on the rheometer flat plate. Strain sweeps were performed at a frequency of 10 rad/s from 0.01% to 1000% strain. Frequency sweeps were performed at 0.01% strain from 1 rad/s to 100 rad/s frequency. All samples were prepared as previously described above in a 2 mL volume in 10.5 mL glass snap cap vials. Rheological data presented in Figures 2, 3 and Figure S2 were obtained from single sample measurements. Rheological data presented in Figure S13 were collected in triplicates and presented as mean ± SD.

**Attenuated Total Reflectance FT-IR Spectroscopy.** Hydrogel samples were prepared as described earlier while just replaced the DI water with deuterium oxide. ATR-FTIR measurements were performed on a Agilent FTIR spectrometer equipped with a diamond ATR accessory. A small bulk of hydrogel was placed on the surface of the diamond and pressed in position using a spatula to ensure good contact between the hydrogel and the diamond surface. The spectra were 32 scans collected with a 2 cm^–1^ resolution.

**Fiber X-ray diffraction.** 10 μL from the hydrogel solutions was placed between two wax-tipped capillary tubes as previously described^2^ and allowed to dry overnight. The resulting air-dried fibre sample was placed on a goniometer head and data were collected on a Bruker D8 Venture MetalJet system using Ga Kα radiation (λ = 1.34138). Data was inspected using Adxv.

**Transmission electron microscopy.** 4μL of hydrogel solution was placed on 400mesh formvar-carbon coated copper grids (Agar Scientific), blotted, the washed with filtered distilled milliQ water, blotted again and then stained with 2% w/v uranyl acetate and finally blotted and allowed to airdry. Electron micrographs were collected JEOL JEM1400-Plus Transmission Electron Microscope operated at 120 kV equipped with a Gatan OneView camera (4k × 4k). Images were recorded at 25 fps with drift correction using GMS3.

**Cryo-EM.** Gel samples were vitrified on lacey carbon grids for the cryo-EM. The surface of the lacey carbon grid was made hydrophilic, prior to the sample application, by glow-discharging (current: 25 mA, duration: 25 seconds) using a GloQube (Quorum Technologies). 3 µL of a peptide sample (concentration: 5 mg/mL, 4x-6x diluted by 7.0 pH water wherever needed) was applied to the glow-discharged lacey carbon grid (Ted Pella INC, 01895-F, 300 mesh, Copper, grid hole size ~63 µm). The excess sample was removed by blotting (Blot force: 6; Blot time: 6 seconds; Wait time: 0 seconds) from the both side of the grid to form a thin film of sample on the lacey carbon, which was plunge-frozen into liquid ethane using a Vitrobot Mark IV (Thermo Fisher Scientific). was applied to the glow-discharged grid, blotted for 3.5 seconds using Whatman filter paper (1001-055) leaving a thin film of sample on the grid. Grid with thin sample film was plunge-frozen in liquid ethane using a Leica EM GP plunge freezer. Imaging of frozen grids was performed using a 200 keV cryo-electron microscope - Glacios (Thermo Fisher Scientific) equipped with a cryo-autoloader, an XFEG™ electron source, and a Falcon 4 detector, housed at the University of Virginia Molecular Electron Microscopy Core facility.

**Additional Data**

**
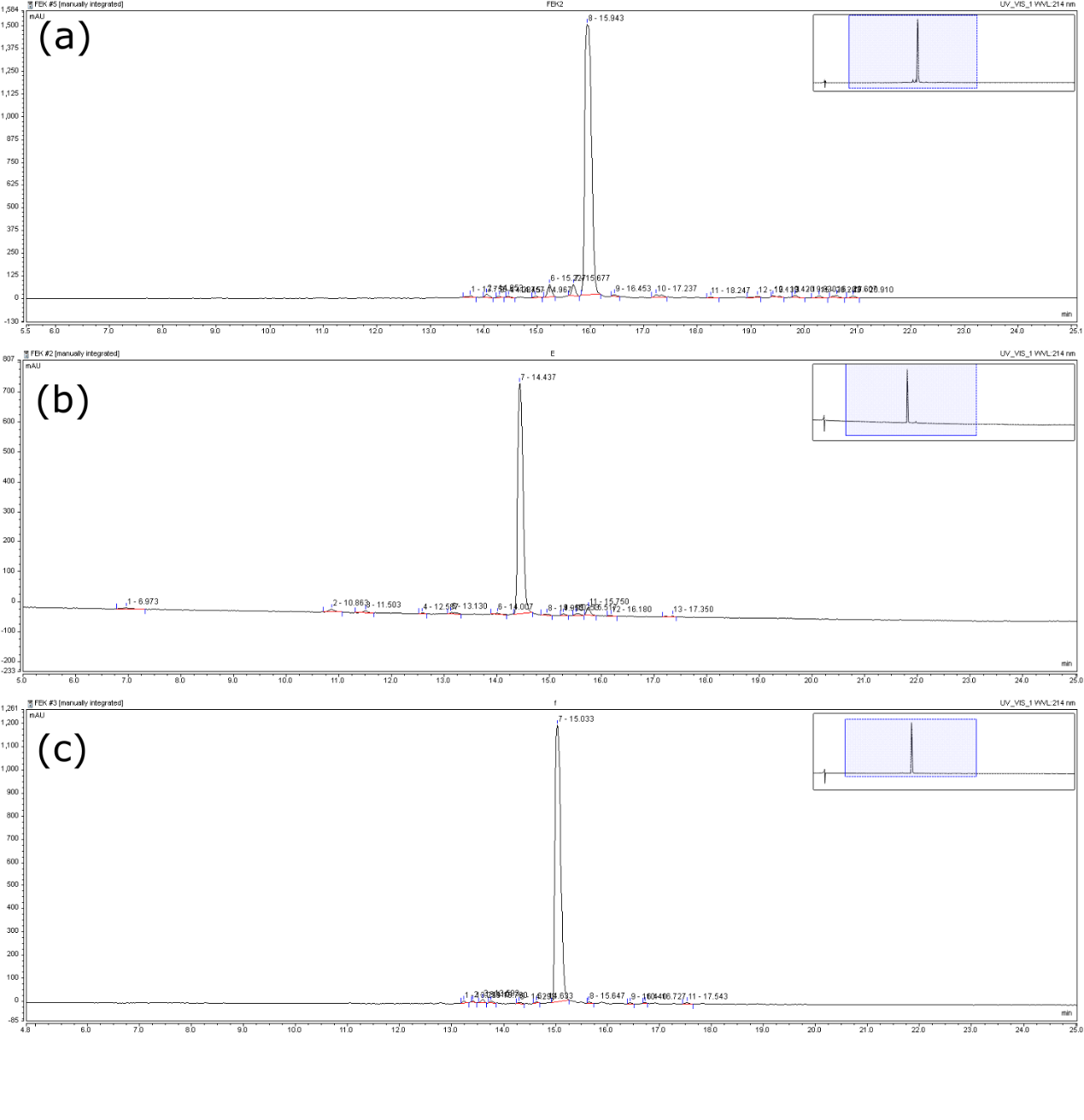
Figure S1.** HPLC of (a) FEFEFKFK; (b) EFEFKFK; (c) FEFEfKFK.

| Peptide | Purity HPLC  (integration at 214nm) | Mass Spectrometry |
| --- | --- | --- |
| FEFEFKFK | 91.3% | Calculated 1121.3, found 1121.5 |
| EFEFKFK | 92.3% | Calculated 974.1, found 974.4 |
| FEFEfKFK | 97.2% | Calculated 1121.3, found 1121.3 |

**Table S1:** HPLC purities and mass spectrometry details for peptides used in this study

**
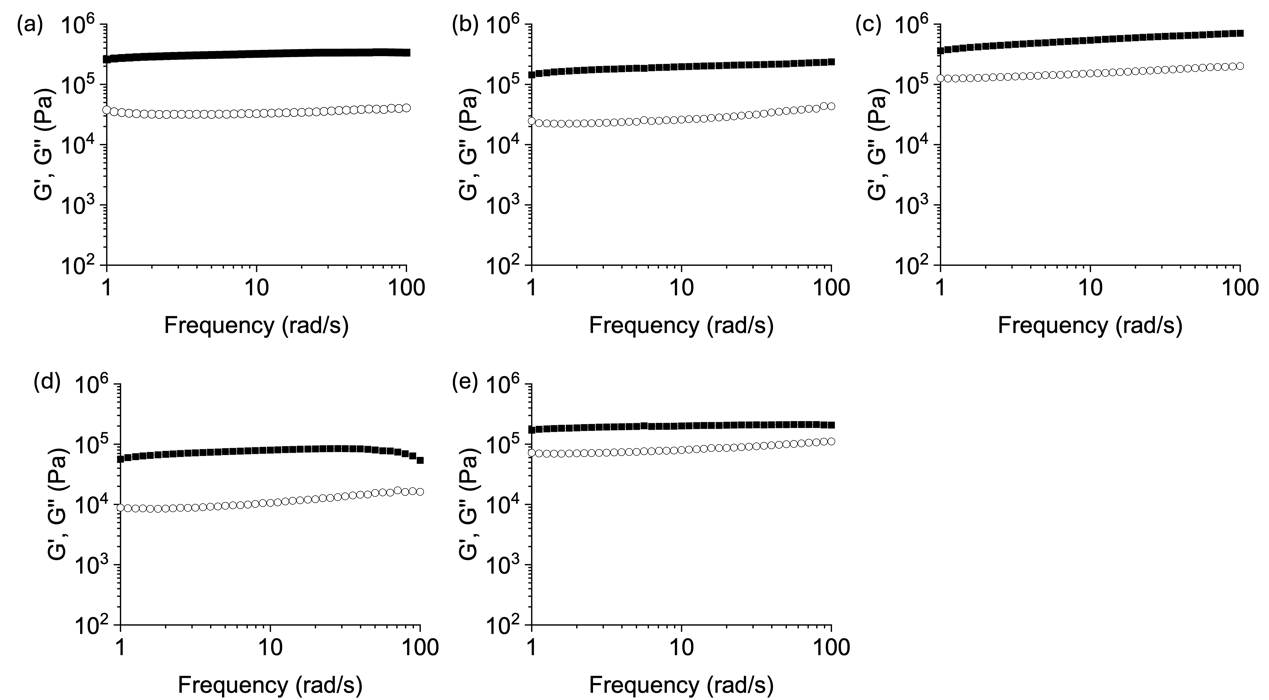
Figure S2.** Frequency sweeps for gels formed from (a) FEFEFKFK; (b) EFEFKFK; (c) FEFEfKFK; (d) 50/50 mixture of FEFEFKFK and EFEFKFK; (e) 50/50 mixture of FEFEFKFK and FEFEfKFK. All gels formed at a total peptide concentration of 20 mg/mL.

**
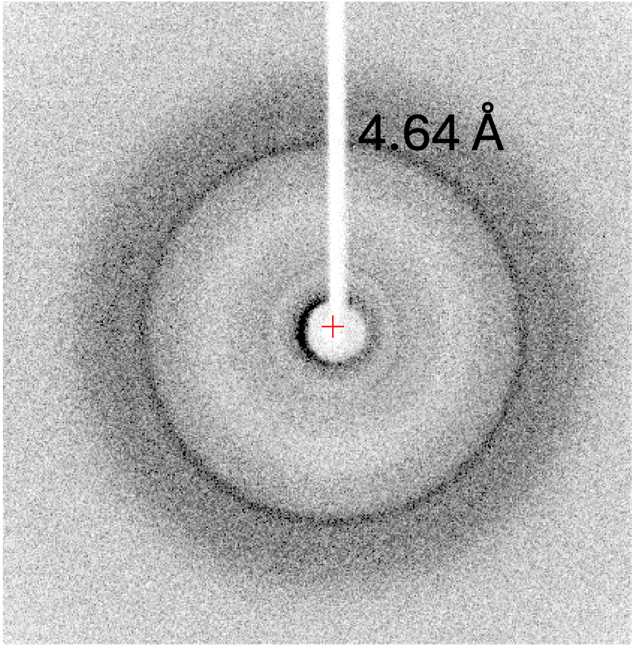
**

**Figure S3.** fXRD for a gel of FEFEFKFK shows a ring with a spacing of 0.46 nm.

**
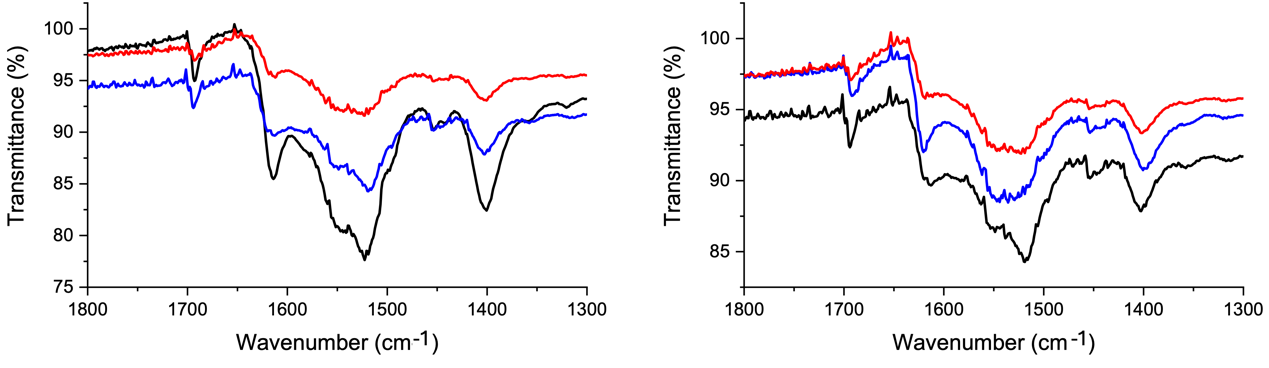
Figure S4.** IR spectra of (left) FEFEFKFK (blue), EFEFKFK (black) and a 50/50 mixture of FEFEFKFK and EFEFKFK (red); (right) FEFEFKFK (blue), FEFEfKFK (black) and a 50/50 mixture of FEFEFKFK and FEFEfKFK (red). All gels formed at a total peptide concentration of 20 mg/mL, with water removed as a background.

**
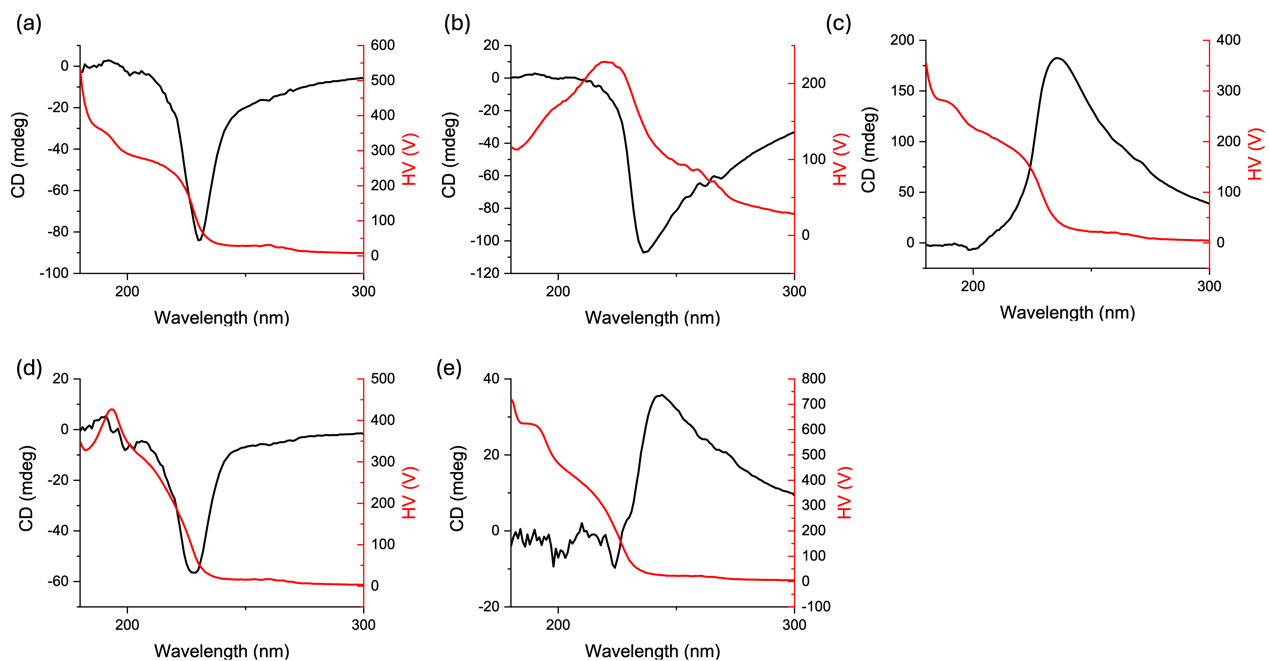
Figure S5.** CD spectra overlaid with HT data. (a) FEFEFKFK; (b) EFEFKFK; (c) FEFEfKFK; (d) a 50/50 mixture of FEFEFKFK/EFEFKFK; (e) a 50/50 mixture of FEFEFKFK/FEFEfKFK. In all cases, the CD data are shown in black (left axis) and the HT data are shown in red (right axis). All gels formed at a total peptide concentration of 20 mg/mL, with water removed as a background.

**
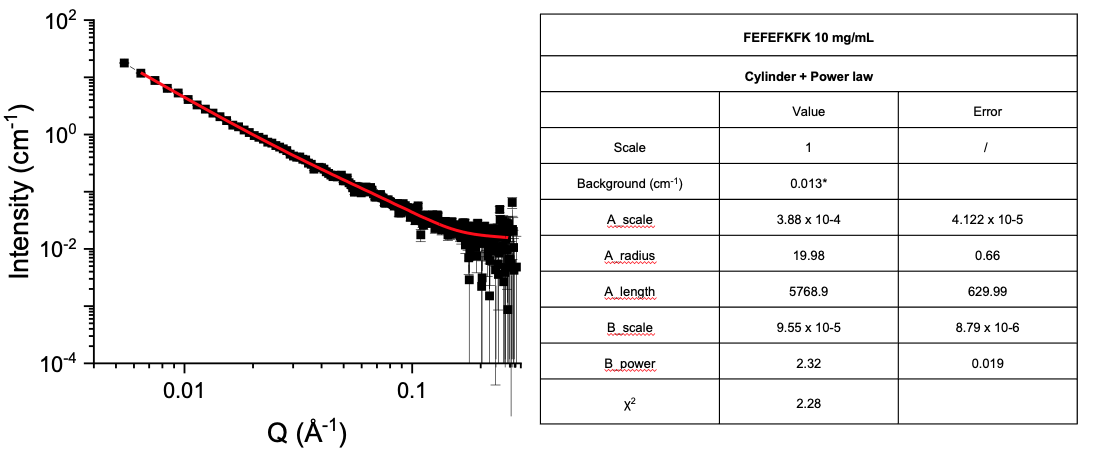
 Figure S6.** SAXS data (black circles), fit (red line) and table showing fit model used and parameters from fit for FEFEFKFK.

**
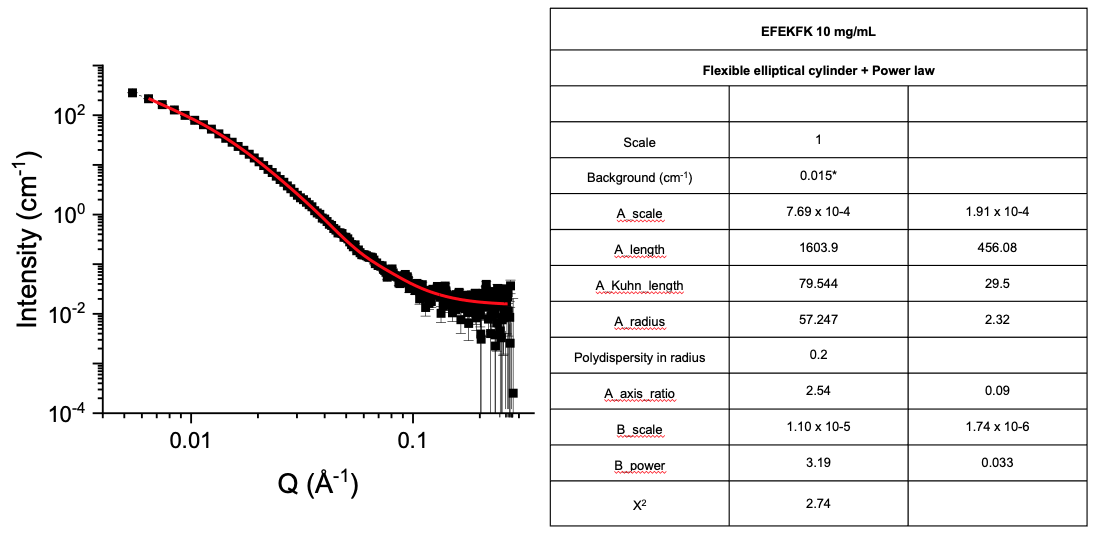
 Figure S7.** SAXS data (black circles), fit (red line) and table showing fit model used and parameters from fit for EFEFKFK.

**
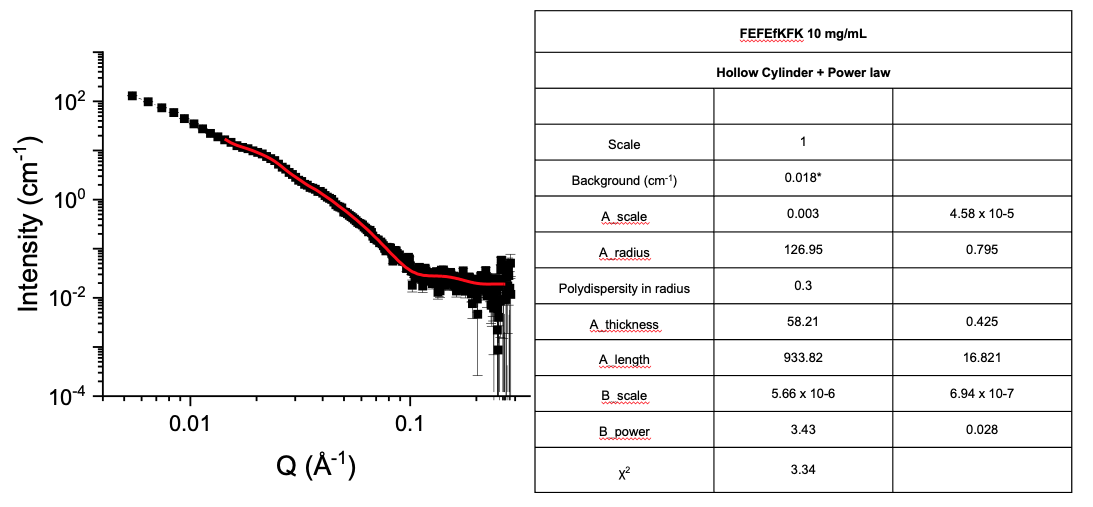
 Figure S8.** SAXS data (black circles), fit (red line) and table showing fit model used and parameters from fit for FEFEfKFK.

**
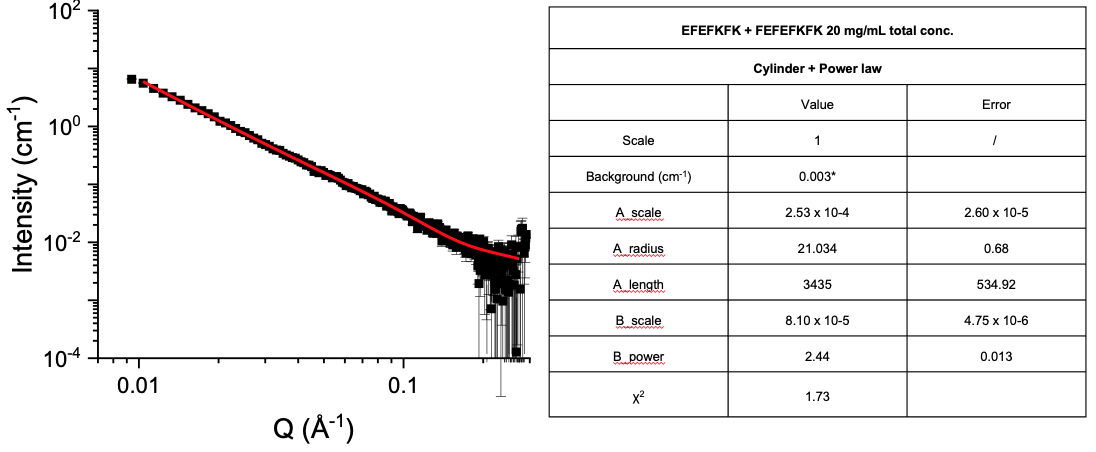
 Figure S9.** SAXS data (black circles), fit (red line) and table showing fit model used and parameters from fit for a 50:50 mixture of FEFEFKFK and EFEFKFK.

**
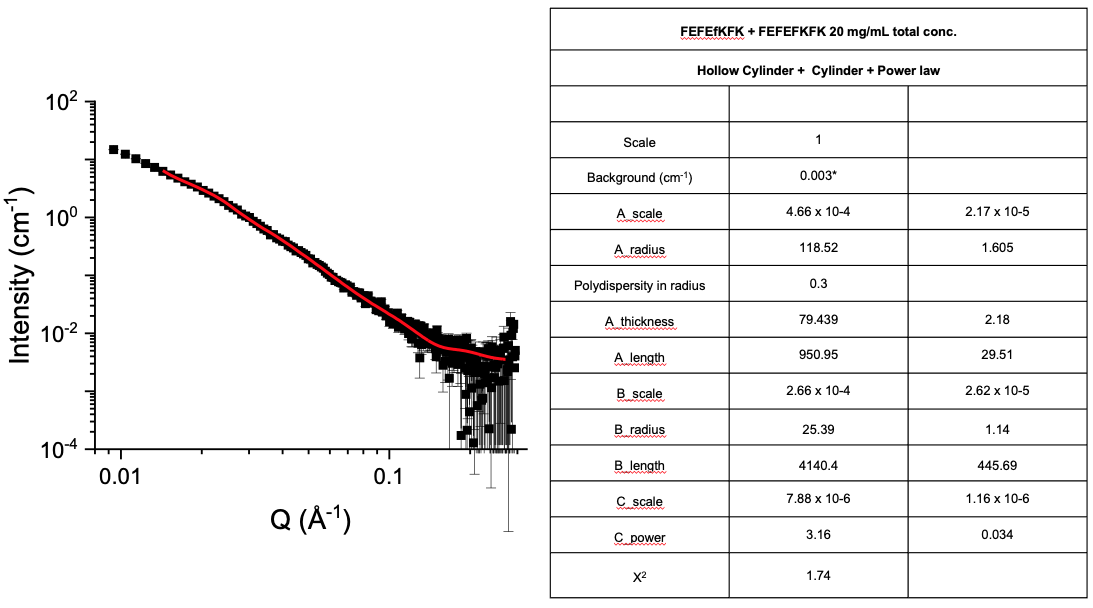
 Figure S10.** SAXS data (black circles), fit (red line) and table showing fit model used and parameters from fit for a 50:50 mixture of FEFEFKFK and FEFEfKFK.


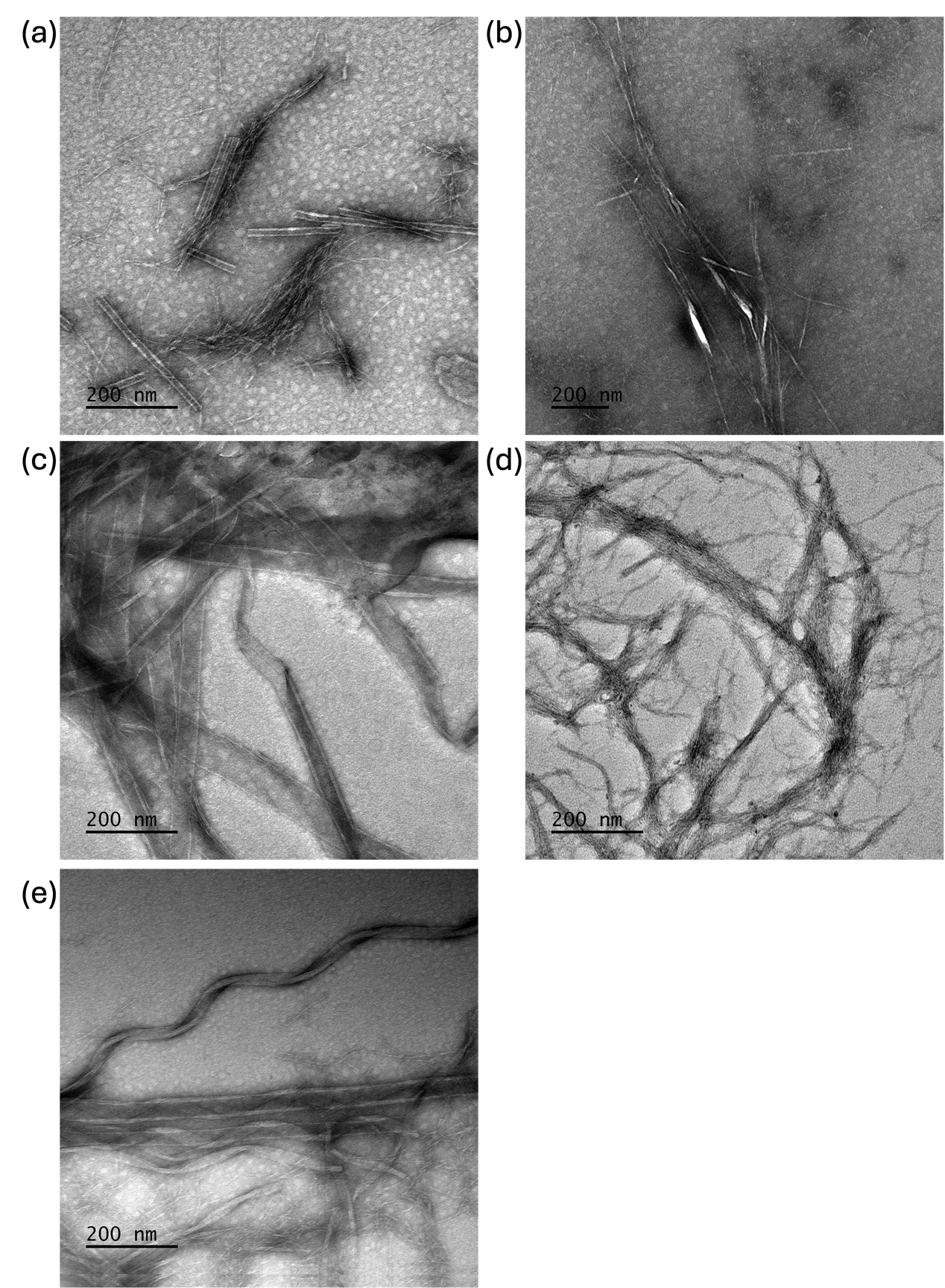


**Figure S11.** TEM data for (a) FEFEFKFK, (b) EFEFKFK (c) FEFEfKFK, (d) 50:50 mix of FEFEFKFK and EFEFKFK and (e) 50:50 mix of FEFEFKFK and FEFEfKFK.


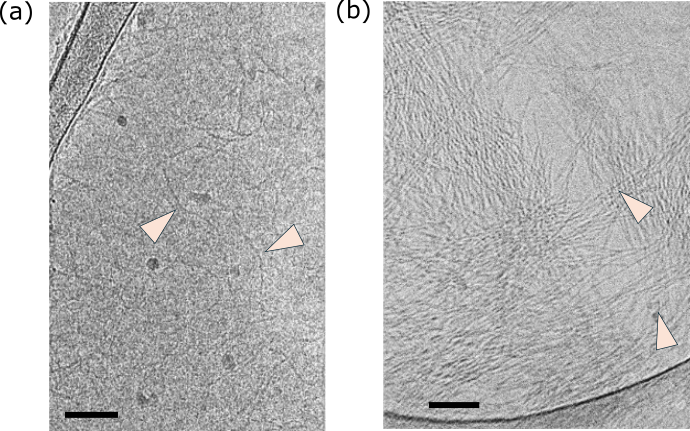


**Figure S12**. Cryo-EM data for diluted samples of (a) FEFEFKFK and (b) EFEFKFK. Scale bars = 50 nm

**
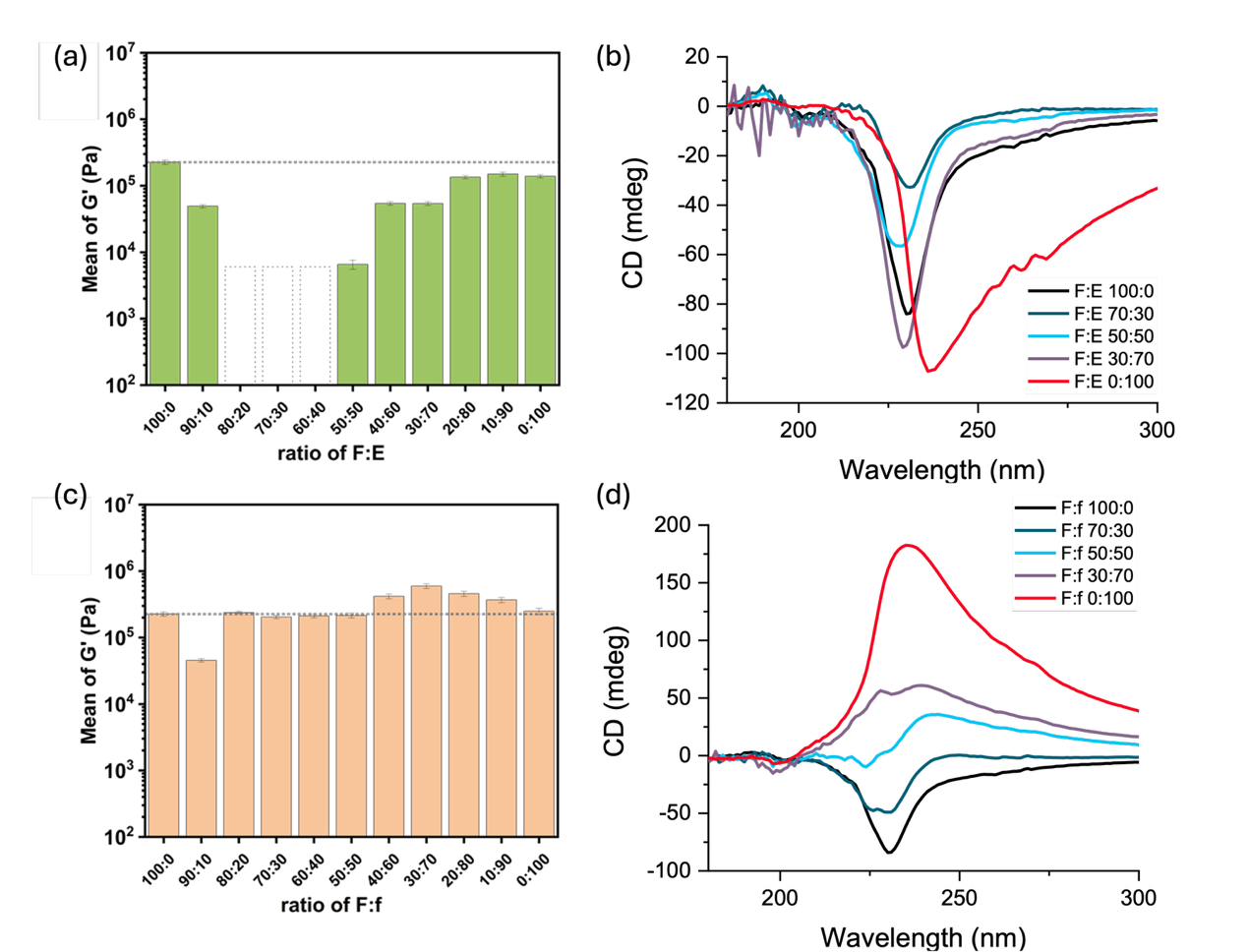
**

**Figure S13.** (a) Storage modulus of gels formed at different ratios of FEFEFKFK to EFEFKFK. Full gelation does not occur at ratios of 80:20, 70:30 and 60:40; (b) CD data for (black) FEFEFKFK, (red) EFEFKFK, (green) a 70:30 mixture of FEFEFKFK and EFEFKFK, (cyan) a 50:50 mixture of FEFEFKFK and EFEFKFK, and (purple) a 30:70 mixture of FEFEFKFK and EFEFKFK; (c) Storage modulus of gels formed at different ratios of FEFEFKFK to FEFEfKFK; Rheological data were collected in triplicates and presented as mean ± SD. (d) CD data for (black) FEFEFKFK, (red) FEFEfKFK, (green) a 70:30 mixture of FEFEFKFK and FEFEfKFK, (cyan) a 50:50 mixture of FEFEFKFK and FEFEfKFK, and (purple) a 30:70 mixture of FEFEFKFK and FEFEfKFK. Circular dichroism spectra were recorded in quadruplicate and averaged. These were recorded as a single measurement on the same sample. The data represents the averaged spectra.

**References**

(1) [www.sasview.org](file:///C:\Users\dave.adams\Library\Containers\com.apple.mail\Data\Library\Mail%20Downloads\C1080D89-2B43-4323-88B5-CBA3C6A08566\www.sasview.org) (accessed 10^th^ August 2024).

(2) Morris, K. L.; Serpell, L. C. X-Ray Fibre Diffraction Studies of Amyloid Fibrils. In *Amyloid Proteins: Methods and Protocols*, Sigurdsson, E. M., Calero, M., Gasset, M. Eds.; Humana Press, 2012; pp 121-135.
